# ChatGPT-4 Turbo and Meta’s LLaMA 3.1: A Relative Analysis of Answering Radiology Text-Based Questions

**DOI:** 10.7759/cureus.74359

**Published:** 2024-11-24

**Authors:** Mohammed Abdul Sami, Mohammed Abdul Samad, Keyur Parekh, Pokhraj P Suthar

**Affiliations:** 1 Department of Diagnostic Radiology and Nuclear Medicine, Rush University Medical Center, Chicago, USA; 2 Department of Osteopathic Medicine, Des Moines University College of Osteopathic Medicine, West Des Moines, USA

**Keywords:** ai in medical education, chatgpt, large language models (llms), llama, pediatric radiology

## Abstract

Aims and objectives: This study aimed to compare the accuracy of two AI models - OpenAI's GPT-4 Turbo (San Francisco, CA) and Meta's LLaMA 3.1 (Menlo Park, CA) - when answering a standardized set of pediatric radiology questions. The primary objective was to evaluate the overall accuracy of each model, while the secondary objective was to assess their performance within subsections.

Methods and materials: A total of 79 text-based pediatric radiology questions were selected out of 302 total questions for this comparison. The questions covered seven subsections, including musculoskeletal, chest, and neuroradiology, among others. Image-based questions were excluded to focus on text interpretation and to minimize the sampling bias within each model. Each model was tested independently on the same question set, and the percent accuracy was calculated for both overall performance as well as individual subsections.

Results: GPT-4 Turbo performed at an overall accuracy of 88.6% (70/79 questions), outperforming LLaMA 3.1’s 77.2% (61/79). Within subsections, GPT-4 Turbo had higher accuracy in most areas, except for equal accuracy in the neuroradiology section. The subsections with the greatest accuracy for GPT-4 Turbo, in descending order, were chest and cardiac radiology (100%), musculoskeletal system (93.3%), and genitourinary system (92.9%). LLaMA 3.1’s highest performance was 86.7% in the musculoskeletal system, while its lowest was 50.0% in chest radiology.

Conclusion: GPT-4 Turbo consistently outperformed LLaMA 3.1 in answering pediatric radiology questions, both overall and within most subsections. These findings suggest that GPT-4 Turbo may offer more accurate responses in specialized medical education, in contrast to LLaMA 3.1’s efficient performance, although future research should further evaluate AI models’ performance within other fields.

## Introduction

Over the past few years, large language models (LLMs) - artificial intelligence (AI) systems trained on deep learning algorithms - have significantly developed and become increasingly sophisticated. These AI systems, such as OpenAI's GPT-4 Turbo (San Francisco, CA) and Meta's LLaMA 3.1 (Menlo Park, CA), utilize techniques to create human-like text responses [1,2]. Their rapid development has transformed various industries, allowing for tasks like diagnostic problem-solving, language translation, and personalized recommendations, showcasing the potential of AI in the future.

Specifically, LLMs, such as GPT-4 Turbo and LLaMA 3.1, both released in 2023, have so far been the foundation of this potential [1-3]. Each model uses similar deep learning analyses based on extensive datasets, accomplishing a wide variety of tasks from generating simple text to complex reasoning. GPT-4 Turbo, an advanced version of GPT-4.0, GPT-3.5, and GPT-3.0, distinguishes itself with its improved “context window,” which means it is more capable of remembering and processing multiple pages of input to generate a more complex output than prior generations [1,4]. Although similar, Meta's LLaMA 3.1 is better known for its efficiency and performance optimization, while GPT-4 Turbo is known for its larger processing capabilities and multimodal task processing [2,4,5].

This study aims to compare these models within a pediatric radiology question set, assessing their capabilities to answer specialized questions accurately. Ultimately, this analysis hopes to delineate each model’s strengths and potential areas for improvement within medical education.

## Materials and methods

This study compared the accuracy of ChatGPT-4 Turbo and Meta’s LLaMA 3.1 by evaluating their responses to 79 text-based questions from the textbook “Pediatric Imaging: A Core Review” [6]. The primary objective was to assess overall accuracy with a secondary objective to analyze each AI model's performance within specific subsections. From the book’s initial pool of 302 questions, 79 were selected based on predetermined criteria that excluded image-based questions. The questions were separated into seven subsections, corresponding with the text’s various focuses such as musculoskeletal and chest radiology, with 38 questions being slightly revised to maintain the consistency of the text-only format.

**Figure 1:**
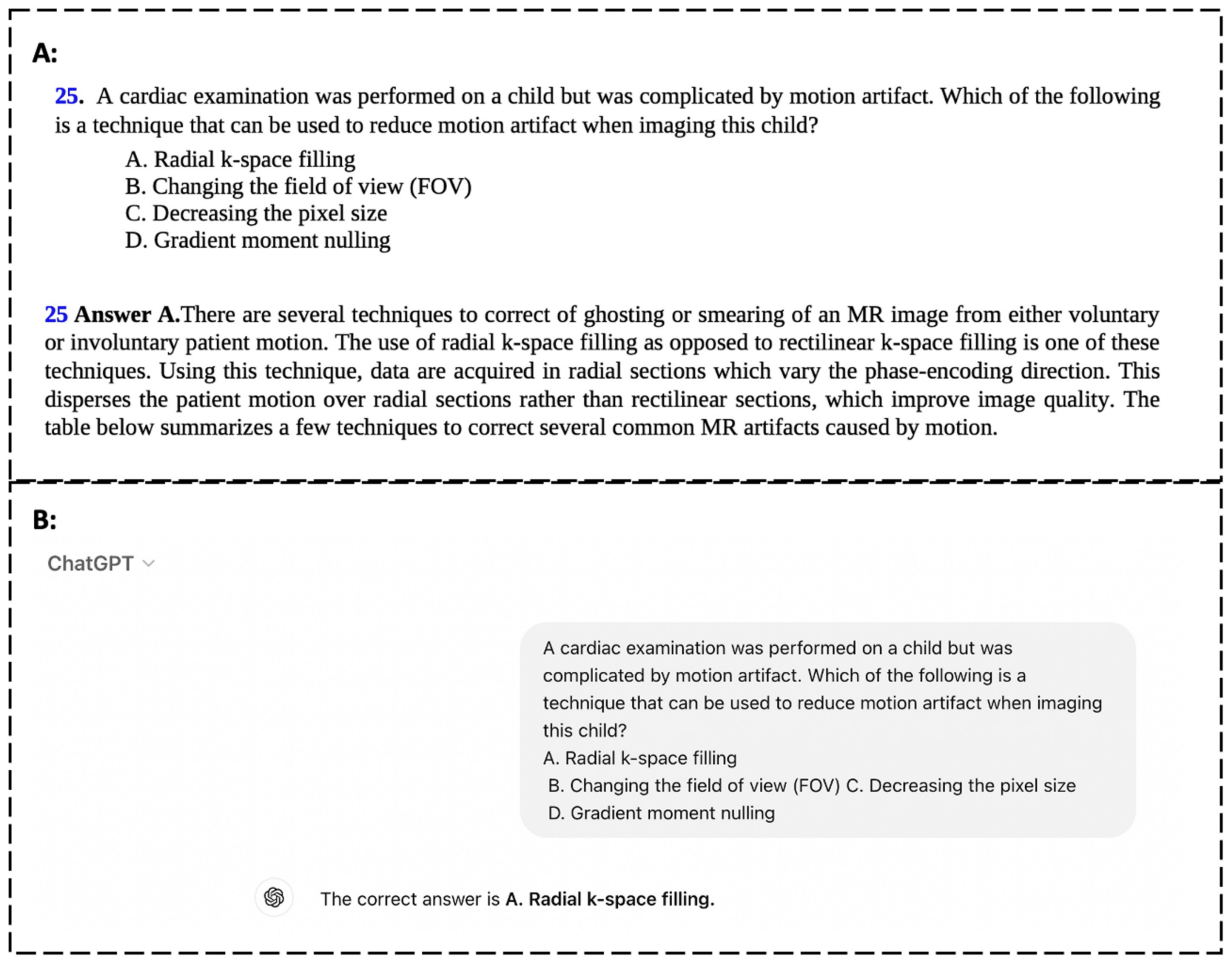
Comparison of responses to a sample textbook question. (A) Part A reflects the original question and answer of one example question as provided in "Pediatric Imaging: A Core Review, 2nd edition," by Blumer et al. [6]. (B) Part B reflects ChatGPT-4 Turbo’s response to the identical question after being presented with four answer choices. This methodology was consistently applied to all eligible questions from this textbook.

**Figure 2:**
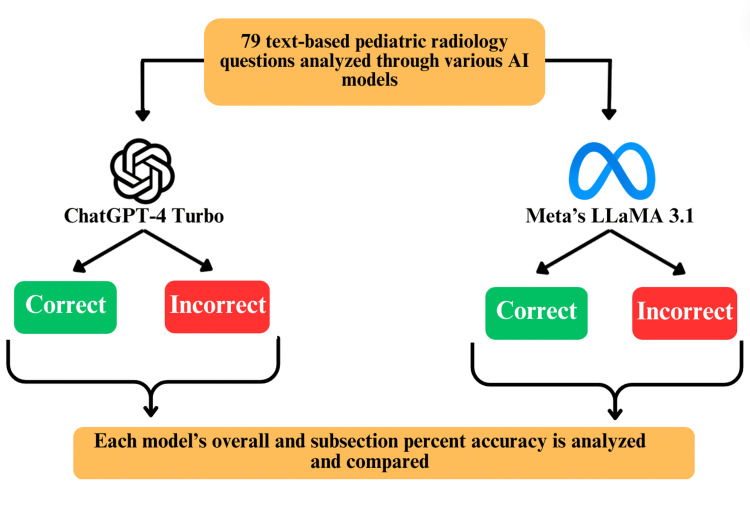
Sequence of methodology to compare ChatGPT-4 Turbo and Meta’s LLaMA 3.1. AI: artificial intelligence.

Each LLM’s accuracy was measured by calculating the percentage of correct answers for each model across the entire question set as well as within subsections. Each model was primed to await a series of multiple-choice questions and to carefully select what they analyzed to be the correct answer (Figure 1). Using a binary answering system, questions answered correctly were notated separately as “1” and incorrectly as “0,” and the final analysis utilized the sum of the individual notations. Analysis of each performance allowed this study to highlight any differences in accuracy, both overall and within subsection (Figure 2). This approach allowed for a clear comparison of how GPT-4 Turbo and LLaMA 3.1 handled pediatric radiology questions. This study did not require Institutional Review Board (IRB) approval due to the lack of pertinent patient data, and all data were cross-checked for diagnostic accuracy.

## Results

**Figure 3:**
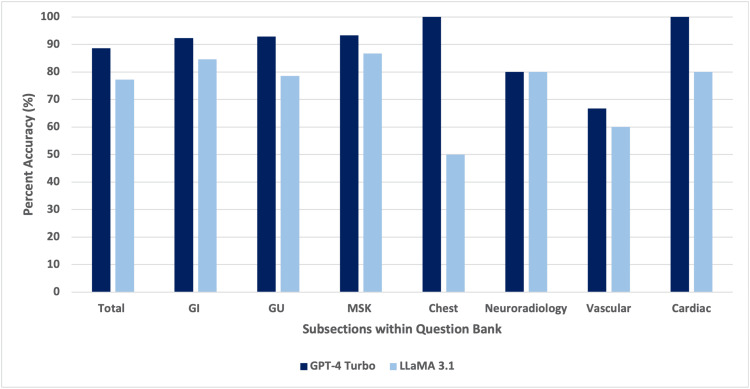
Bar graph illustrating differences in performance between ChatGPT-4 Turbo and LLaMA 3.1. GI: gastrointestinal; GU: genitourinary; MSK: musculoskeletal.

**Figure 4:**
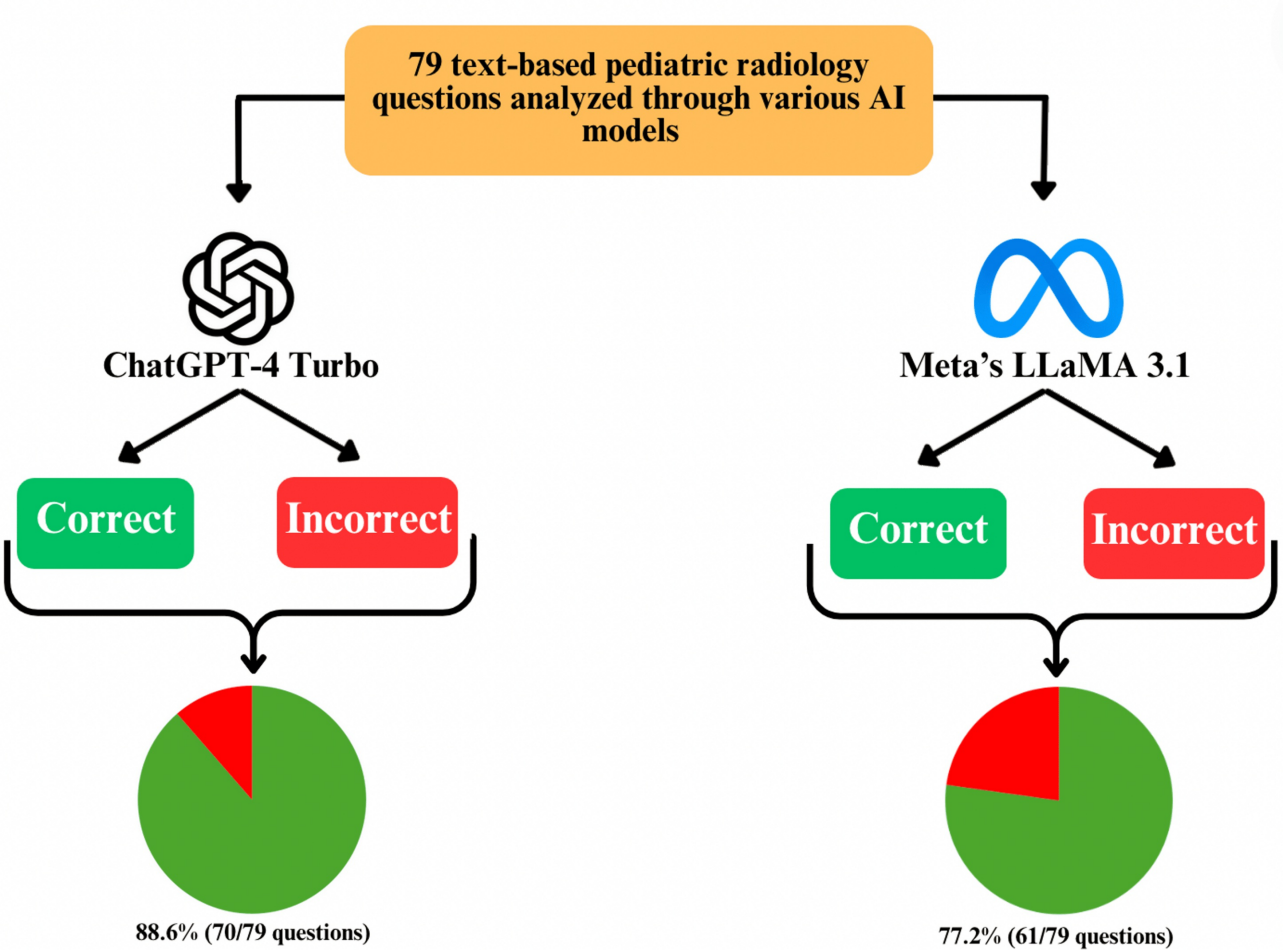
Results of comparison and percent accuracies between ChatGPT-4 Turbo and LLaMA 3.1. AI: artificial intelligence.

In this analysis, GPT-4 Turbo demonstrated a greater overall accuracy compared to Meta's LLaMA 3.1. GPT-4 Turbo correctly answered 88.6% of the 79 questions (70/79), while LLaMA 3.1 correctly answered 77.2% (61/79). When the results were further divided into subsections, GPT-4 Turbo consistently outperformed LLaMA 3.1 in most areas (Figures 3, 4).

GPT-4 Turbo performed at 92.3% (12/13) in the gastrointestinal system, 92.9% (13/14) in the genitourinary system, 93.3% (14/15) in the musculoskeletal system, 100% (2/2) in chest radiology, 80.0% (4/5) in neuroradiology, 66.7% (10/15) in vascular radiology, and 100% (15/15) in cardiac radiology. In comparison, LLaMA 3.1 scored 84.6% (11/13) in the gastrointestinal system, 78.6% (11/14) in the genitourinary system, 86.7% (13/15) in the musculoskeletal system, 50% (1/2) in chest radiology, 80.0% (4/5) in neuroradiology, 60.0% (9/15) in vascular radiology, and 80.0% (12/15) in cardiac radiology (Table 1).

**Table 1:** Comparative percent accuracies between ChatGPT-4 Turbo and LLaMA 3.1. GI: gastrointestinal; GU: genitourinary; MSK: musculoskeletal.

	Number of questions	GPT4-Turbo's percent accuracy (%)	Number of questions, correct/total	LLaMA 3.1's percent accuracy (%)	Number of questions, correct/total
Overall	79	88.6%	70/79	77.2%	61/79
GI tract	13	92.3%	12/13	84.6%	11/13
GU tract	14	92.3%	13/14	78.6%	11/14
MSK	15	93.3%	14/15	86.7%	13/15
Chest	2	100.0%	2/2	50.0%	1/2
Neuroradiology	5	80.0%	4/5	80.0%	4/5
Vascular	15	66.7%	10/15	60.0%	9/15
Cardiac	15	100.0%	15/15	80.0%	12/15

In terms of specific accuracy by subsection, GPT-4 Turbo performed best in chest radiology and cardiac radiology (100.0%), followed by the musculoskeletal system (93.3%), gastrointestinal system (92.3%), and genitourinary system (92.9%). The lowest performance for GPT-4 Turbo was in vascular radiology, at 66.7%. LLaMA 3.1's highest accuracy was in the musculoskeletal system (86.7%), and its lowest was in chest radiology (50.0%).

## Discussion

Overall & subsection performance per AI model

GPT-4 Turbo’s greater percent accuracy compared to LLaMA 3.1 initially implies its better processing of text-based questions. This performance could likely be attributed to its training on larger, more specialized datasets, which are better equipped for answering specific questions, unlike LLaMA 3.1, which was developed to handle a broader research focus with greater efficiency [1,6]. As stated previously, GPT-4 Turbo's greater context window, which enhances its level of complex problem-solving, may strengthen its ability to handle complex questions [1,2].

Of note, the similar percentage accuracies in smaller categories such as neuroradiology and vascular radiology are likely due to the limited number of text-based questions compared to larger sections. The study's exclusion of image-based questions, which do not mirror a realistic radiology assessment, may have restricted the scope of our research as well.

General strengths and limitations of AI in medicine

AI offers tools and the integration of its ever-growing database that continues to manipulate data more quickly and sometimes more accurately than humans [6,7]. In specific medical fields, such as radiology, these LLMs have the potential to help with quickly interpreting the literature on patient conditions, summarizing patient histories, or creating reports. In fact, prior comparative studies between earlier versions of these LLMs have found significant overall differences in accuracy, which may be a byproduct of each LLM’s intended function [8-10]. However, concerns remain about the ability of these models to correctly interpret and analyze images or provide the correct information, especially with reported events in the past of incorrect analyses [7,11]. While the rise of these models has been rapid, their integration in most systems is still in its growing stages, and analyses such as this study continue to mark its ever-growing capabilities and potential for the future.

One interesting note between GPT-4 Turbo and Meta’s LLaMA 3.1 is the difference between open-source software and proprietary models [1]. Currently, LLaMA is labeled as open source, which allows developers to openly create or experiment with the model’s strengths and limitations. However, GPT-4 Turbo is labeled as a “proprietary” model, which is aimed toward commercial use [1]. Customers are still allowed to use its tools and database, but this limits the allowable experimentation compared to LLaMA [1,4]. This dichotomy between the models may be a talking point in the future if one model becomes more accessible to change, such as in rapidly evolving fields like medicine.

Public perception and trust in AI for healthcare

The ethical consideration of utilizing AI in healthcare fields is complex and significantly intersects with the public perception of AI in general. There is clearly growing excitement for the future of these LLMs within multiple fields, and many view these models with optimism that they may improve the speed of various tasks and reduce human error [12,13]. However, problems arise around the accuracy and acceptability of AI's decision-making, especially when used in urgent situations or given that certain models may only be trained on limited datasets [12-14]. Specifically in medicine, many studies report that patients look for trust in their judgment of AI models and that these models are not currently at that level yet [13-16]. This discrepancy is further noted in this study, where different AI models are seen to have differing performances within the same question set, further emphasizing the lack of standardized training across models [16,17]. Each of these models is trained with specific tasks in mind and while they will continue to improve over decades, their extensive training is yet to be developed in fields where precision is demanded, such as radiology.

Future direction

In the future, studies incorporating image-based questions would be a useful additive to realistically compare each model’s true capabilities, especially considering GPT-4 Turbo’s improved context windows. Additional models may also be compared such as Microsoft Copilot, Claude 3 Opus, or others with GPT-4 Turbo or LLaMA 3.1, which would better assess the ability of each model within different aspects of medicine and education [17]. Exploring the abilities of these AI models in diagnostic scenarios specifically could offer insight into their practical use in fields like radiology, and following up with longitudinal studies could help observe the evolution of these models' performances over time.

## Conclusions

The findings of this study reflect a greater percent accuracy with GPT-4 Turbo compared to LLaMA 3.1 in answering a standardized set of pediatric radiology questions, with GPT-4 Turbo performing at 88.6% correct (70/79) compared to LLaMA 3.1's 77.2% (61/79). These data suggest that GPT-4 Turbo may have an improved dataset for handling specialized text-based tasks. However, the variance across different subsections, such as neuroradiology, was minimal, and statistical significance was not comparably assessed. These findings highlight the need for cautious interpretation of the data and suggest that both models have unique strengths and limitations that should be considered when integrating AI in various settings.

In conclusion, this study underscores the potential of AI models in medical education, especially in specialized fields such as radiology where accurate interpretations of images are critical. As AI continues to evolve, it offers exciting potential to medicine, radiology, and overall patient outcomes. However, it is vital that these models are utilized ethically and as a supplement to human expertise. Future research should continue to investigate these models to optimize AI while upholding its responsible application.
